# Comparative evaluation of nano-hydroxyapatite–silica modified glass ionomer cement as a luting agent for stainless steel crowns using the Hall technique: An in vitro study

**DOI:** 10.4317/jced.63884

**Published:** 2026-03-30

**Authors:** Fadzlinda Baharin, Siti Nuramira Izzati Che Abdullah, Nik Rozainah Nik Abdullah Ghani, Aimi Kamarudin

**Affiliations:** 1 DR. School of Dental Sciences, UniversitI Sains Malaysia, 16150 Kubang Kerian, Kelantan, Malaysia; 2 Associate Professor. School of Dental Sciences, UniversitI Sains Malaysia, 16150 Kubang Kerian, Kelantan, Malaysia

## Abstract

**Background:**

Nano-hydroxyapatite and silica incorporation into glass ionomer cements (GICs) have been proposed to enhance their physical and biological properties. In paediatric dentistry, the Hall technique relies heavily on the sealing ability of luting cements used for stainless steel crown (SSC) placement. This study aimed to compare the microleakage of nano-hydroxyapatite-silica incorporated glass ionomer cement (nano-HA-silica GIC) with conventional GIC and resin-modified glass ionomer cement (RMGIC) when used as luting agents for SSCs placed using the Hall technique.

**Material and Methods:**

Thirty extracted human primary molars were randomly allocated into three groups (n = 10). Stainless steel crowns were cemented using conventional GIC, nano-HA-silica GIC, or RMGIC following the Hall technique protocol. Specimens were immersed in 2% methylene blue dye, sectioned longitudinally, and evaluated under a digital microscope to assess dye penetration. Microleakage values were statistically analysed using one-way ANOVA followed by the Games-Howell post hoc test.

**Results:**

Statistically significant differences in microleakage were observed among the three groups (p &lt; 0.001). RMGIC demonstrated significantly lower microleakage compared with both conventional GIC (mean difference = 1.568) and nano-HA-silica GIC (mean difference = 2.290). No statistically significant difference was observed between conventional GIC and nano-HA-silica GIC (mean difference = 0.722).

**Conclusions:**

Nano-hydroxyapatite-silica incorporated GIC exhibited comparable sealing ability to conventional GIC; however, RMGIC demonstrated superior resistance to microleakage when used as a luting cement for SSCs placed using the Hall technique. Further studies are required to evaluate the long-term clinical performance of nano-HA-silica modified GIC.

## Introduction

Stainless steel crowns (SSCs) are widely regarded as the restoration of choice for multisurface carious lesions in primary molars because of their durability, longevity, and ability to provide full coronal coverage (1 - 3). The Hall technique is a minimally invasive approach for SSC placement that avoids caries removal, tooth preparation, and local anaesthesia, and has demonstrated high clinical success and acceptability in paediatric dentistry (1 , 4 - 6). The success of SSCs placed using the Hall technique depends largely on the sealing ability of the luting cement at the tooth-crown interface (7 , 8). Microleakage may compromise marginal integrity, allow bacterial penetration, and potentially lead to restoration failure (9 , 10). Therefore, appropriate selection of luting cement is critical for ensuring long-term clinical success. Glass ionomer cements (GICs) are commonly used as luting agents for SSCs due to their chemical adhesion to tooth structure, fluoride release, and ease of manipulation (11 - 13). However, conventional GICs exhibit limitations such as low fracture toughness and susceptibility to dissolution (14 , 15). Resin-modified glass ionomer cements (RMGICs) were developed to address these shortcomings and have demonstrated improved mechanical properties and marginal sealing ability (12 , 13). Recent advances in dental materials research have focused on incorporating nanoparticles such as nano-hydroxyapatite into GICs to enhance their mechanical and physical properties (16 - 21). While laboratory studies have shown improvements in hardness and strength following nanoparticle incorporation, evidence regarding the sealing ability of nano-hydroxyapatite-silica modified GIC when used as a luting agent for SSCs placed using the Hall technique remains limited. Therefore, this in vitro study aimed to compare the microleakage of nano-hydroxyapatite-silica incorporated glass ionomer cement with conventional glass ionomer cement and resin-modified glass ionomer cement when used as luting agents for stainless steel crowns placed using the Hall technique.

## Materials and Methods

- Sample Size Calculation The sample size was determined using G*Power software version 3.1.9.6 (Franz Faul, Universität Kiel, Germany). An F-test family with one-way analysis of variance (ANOVA) was selected as the statistical test. The parameters were set as follows: alpha error probability of 0.05, statistical power (1) of 0.80, and three study groups. Based on the study by Memarpour et al. (8), an effect size (f) of 0.8829787 was calculated. The minimum required sample size was six specimens per group. To increase reliability, a total of ten specimens per group was used, resulting in 30 samples overall. - Sample Selection and Preparation Thirty extracted human primary molars were collected, cleaned, disinfected, and stored in distilled water until use. Thirty extracted human primary molars were collected and stored in distilled water until use. The teeth selected were either sound or had caries limited to two surfaces. The mesiodistal dimension of each tooth was measured using a periodontal probe to determine the appropriate stainless steel crown (SSC) size. No tooth preparation or crown crimping was performed, in accordance with the Hall technique. The smallest prefabricated SSC that could be seated on each tooth was selected using a trial-and-error method. All crowns were cleaned and sandblasted prior to cementation. Commercially available conventional glass ionomer cement (GIC) (Riva Luting, SDI Limited, Germany) and resin-modified glass ionomer cement (RMGIC) (GC Fuji Plus, GC Corporation, Japan) were used as control luting agents. The experimental luting agent was prepared by incorporating 1% nano-hydroxyapatite-silica (nHA-SiO2) particles into the glass ionomer cement. The materials used in this study were summarised in Table 1.

Table 1

**Figure 1:**
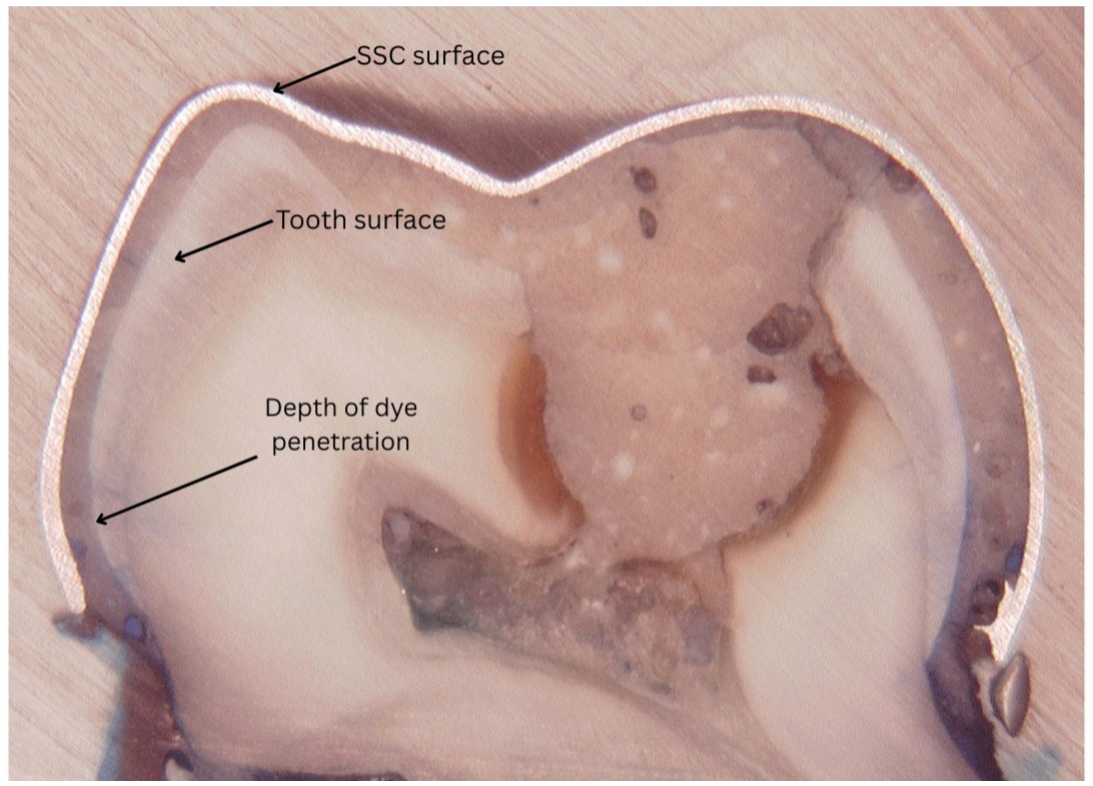
Longitudinal section of a primary molar with a stainless steel crown (SSC), showing dye penetration used to assess microleakage at the tooth-crown interface.

- Grouping and Cementation Procedure The prepared teeth were randomly assigned into three groups (n = 10 per group). Group 1 specimens were cemented using conventional GIC, Group 2 using nano-hydroxyapatite-silica incorporated GIC, and Group 3 using resin-modified GIC. All luting agents were mixed according to the manufacturers' instructions and placed inside the crowns. The crowns were seated onto the teeth using finger pressure, and excess cement was removed. The specimens were then stored in distilled water at 37°C and 100% humidity for 24 hours. - Microleakage Evaluation For microleakage assessment, the specimens were immersed in 2% methylene blue dye solution (MKCD3437, Sigma-Aldrich, USA) for 24 hours. Following dye immersion, the specimens were rinsed with distilled water, air-dried, and embedded in epoxy resin. Each sample was sectioned longitudinally in a buccolingual direction using a hard tissue cutter (EXAKT 312, EXAKT Technologies Inc., Oklahoma City, USA). Dye penetration was evaluated using a digital microscope (HiRoX KH-7700, HiRoX Co. Ltd., Japan) under 50× magnification (Fig. 1).

1

The examiner assessing dye penetration was blinded to the group allocation. - Statistical Analysis Descriptive statistics, including mean and standard deviation, were calculated for microleakage values in all groups. Differences in microleakage among the three luting cements were analysed using one-way ANOVA followed by the Games-Howell post hoc test. Statistical analysis was performed using the Statistical Package for the Social Sciences (SPSS) version 20.0 (IBM Corp., Chicago, USA). The level of statistical significance was set at p &lt; 0.05.

## Results

**Figure 2:**
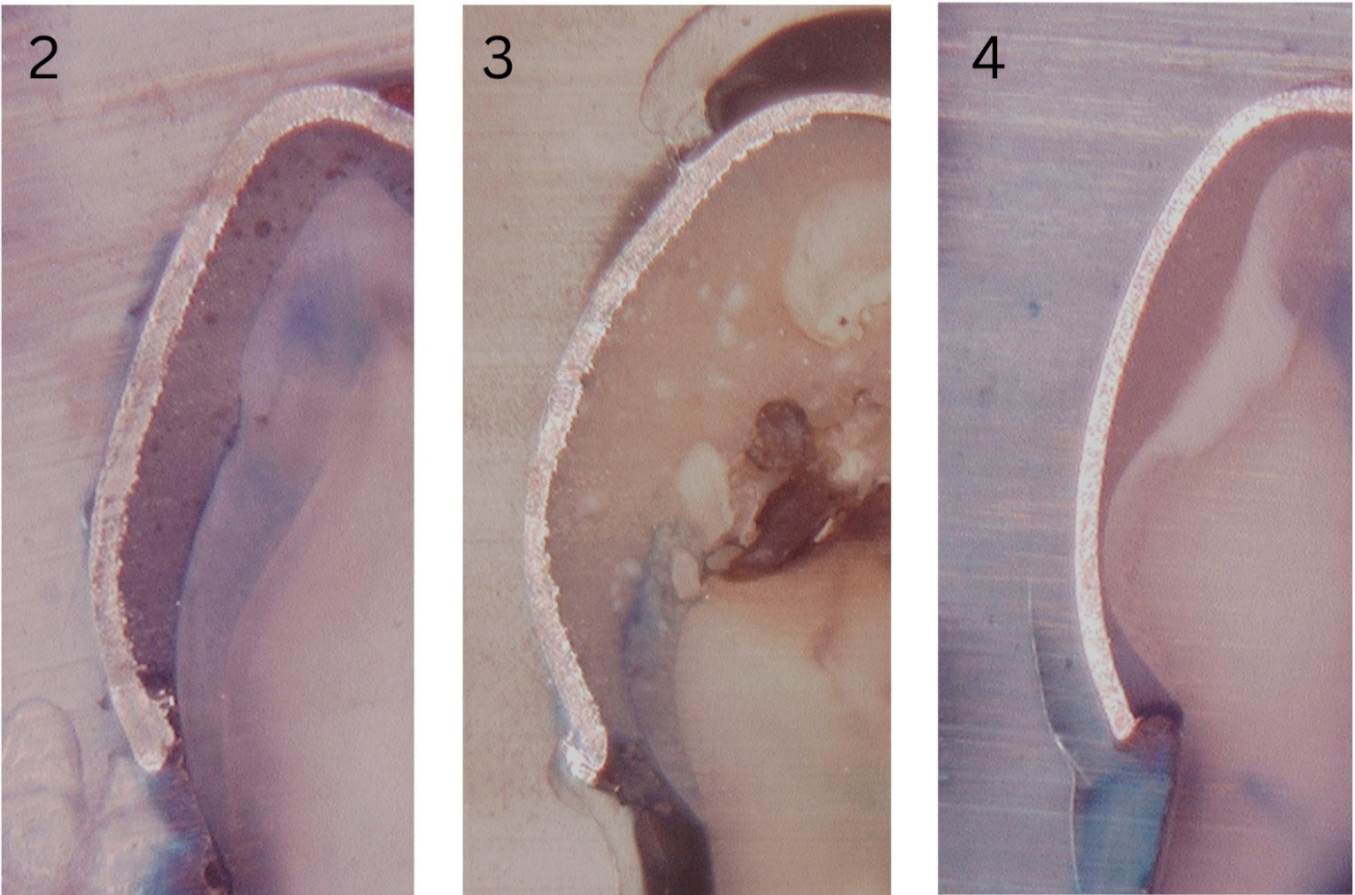
Representative longitudinal sections showing dye penetration (microleakage) at the tooth-crown interface for different luting cements: (2) conventional GIC, (3) nano-hydroxyapatite-silica GIC, and (4) resin-modified GIC.

Representative digital microscope images illustrating microleakage patterns for conventional glass ionomer cement, nano-hydroxyapatite-silica incorporated glass ionomer cement, and resin-modified glass ionomer cement are shown in Fig. 2.

2

Table 2 shows that the highest mean microleakage was observed in the nano-hydroxyapatite-silica GIC group (3.44 ± 2.06 mm), followed by the conventional GIC group (2.72 ± 1.42 mm).

Table 2

The lowest mean microleakage was recorded in the resin-modified GIC group (1.15 ± 0.97 mm). One-way analysis of variance revealed a statistically significant difference in microleakage among the three luting cements (p &lt; 0.001). Multiple comparisons using the Games-Howell post hoc test demonstrated significantly lower microleakage values for resin-modified GIC compared with both conventional GIC (mean difference = 1.568; p &lt; 0.001) and nano-hydroxyapatite-silica GIC (mean difference = 2.290; p &lt; 0.001). No statistically significant difference in microleakage was observed between conventional GIC and nano-hydroxyapatite-silica GIC (mean difference = 0.722; p &gt; 0.05), as shown in Table 3.

Table 3

## Discussion

The present in vitro study evaluated the microleakage of three different luting cements used for stainless steel crown cementation employing the Hall technique. The results demonstrated that resin-modified glass ionomer cement (RMGIC) exhibited significantly lower microleakage compared with conventional glass ionomer cement (GIC) and nano-hydroxyapatite-silica incorporated GIC. Assessment of microleakage using dye penetration is a widely accepted and reliable method for evaluating marginal sealing ability in in vitro studies (10 , 15). Using this method, the superior sealing performance of RMGIC observed in the present study may be attributed to the presence of resin components, which enhance adhesion, reduce solubility, and improve marginal integrity. Similar findings have been reported in previous studies evaluating microleakage of stainless steel crowns cemented with different luting agents (15 , 22 , 23), supporting the results of the present investigation. Although nano-hydroxyapatite-silica modified GIC has demonstrated enhanced mechanical properties such as increased hardness and compressive strength in laboratory-based disc studies (18 - 21), these improvements do not necessarily translate into superior sealing ability when used as a luting cement in extracted teeth. Mechanical testing on standardized specimens does not fully replicate clinical conditions, where adhesion, flow characteristics, and marginal adaptation play a critical role in controlling microleakage (24). The lack of improved sealing observed with nano-modified GIC may possibly be related to nanoparticle agglomeration or alterations in cement viscosity, which could negatively influence adaptation at the tooth-crown interface (19 , 25). In the present study, microleakage assessment provided a more clinically relevant evaluation of luting performance, demonstrating that nano-hydroxyapatite-silica incorporated GIC performed comparably to conventional GIC, while RMGIC provided a superior coronal seal. The Hall technique presents an additional challenge, as stainless steel crowns are placed without tooth preparation or crimping, resulting in inevitable marginal discrepancies (2 , 5). Under such conditions, the luting cement plays a critical role in compensating for gaps and ensuring an adequate coronal seal. RMGIC, with its dual-setting mechanism involving both an acid-base reaction and resin polymerization, exhibits improved flow and adhesion, enabling effective gap filling and marginal adaptation (7 , 8). In contrast, nano-hydroxyapatite-silica modified GIC, despite improved bulk properties, does not appear to significantly enhance marginal sealing at these discrepancies, explaining its performance being similar to that of conventional GIC. This study has certain limitations, including a relatively small sample size and its in vitro design, which may not fully replicate oral conditions such as masticatory forces, saliva exposure, thermal cycling, and long-term aging (10). Future studies incorporating thermomechanical loading and clinical trials are warranted to further evaluate whether nano-hydroxyapatite-silica modifications can enhance the adhesive performance of glass ionomer cements in clinical practice. Overall, while nano-hydroxyapatite-silica incorporated GIC demonstrated sealing ability comparable to conventional GIC, resin-modified glass ionomer cement provided superior resistance to microleakage and remains the preferred luting material for clinical applications requiring reliable coronal sealing, particularly when using the Hall technique.

## Conclusions

Within the limitations of this in vitro study, resin-modified glass ionomer cement demonstrated superior resistance to microleakage compared with conventional glass ionomer cement and nano-hydroxyapatite-silica incorporated glass ionomer cement when used as a luting agent for stainless steel crowns placed using the Hall technique. Nano-hydroxyapatite-silica incorporated glass ionomer cement showed comparable sealing ability to conventional glass ionomer cement. Further clinical studies are recommended to evaluate the long-term performance of nano-modified glass ionomer cements in paediatric dentistry.

## Figures and Tables

**Table 1:** Materials used in the study.

Material	Manufacturer	Type	Composition
GC Fuji Plus	GC Corporation, Japan	Resin-modified glass ionomer cement	Powder: Fluoroaluminosilicate glass, polyacrylic acidLiquid: Polyacrylic acid, polybasic carboxylic acid
Riva Luting	SDI Limited, Germany	Glass ionomer cement	Powder: Glass powderLiquid: Acrylic acid homopolymer
Nano-hydroxyapatite–silica GIC	Universiti Sains Malaysia, Malaysia	Nano-hydroxyapatite–silica incorporated glass ionomer cement	Powder: Calcium hydroxide, phosphoric acid, tetraethyl orthosilicate, ethanol, ammoniaLiquid: Polyacrylic acid, polybasic carboxylic acid

1

**Table 2:** Mean ± standard deviation (SD) of microleakage (mm) for different luting cements.

Luting cements	Microleakage(Mean ± SD)	P-value
Nano-hydroxyapatite-silica GIC	3.44 ± 2.06	<.05
Conventional GIC	2.72 ± 1.42	
Resin-modified GIC	1.15 ± 0.97	

2

**Table 3:** Pairwise comparison of microleakage values (mm) among different luting cements using the Games–Howell post hoc test.

Luting cements		Mean difference	P-value	95% Confidence Interval	
				Lower	Upper
Conventional GIC	Resin-modified GIC	1.568	<0.001*	0.915	2.222
Nano-hydroxyapatite-silica GIC		2.290	<0.001*	1.420	3.160

Pairwise comparison of microleakage values (mm) among different luting cements using the Games–Howell post hoc test.

## Data Availability

Available upon request.
